# Study on Quantitative Adjustment of CD Bias and Profile Angle in the Wet Etching of Cu-Based Stacked Electrode

**DOI:** 10.3390/ma18010116

**Published:** 2024-12-30

**Authors:** Dan Liu, Liang Fang, Zhonghao Huang, Jianguo An, Xu Wu, Fang Wu, Wenxiang Chen, Gaobin Liu

**Affiliations:** 1 Chongqing Key Laboratory of Interface Physics in Energy Conversion, College of Physics, Chongqing University, Chongqing 400044, China; ld54400398@163.com (D.L.); wufang@cqu.edu.cn (F.W.); otforcwx@outlook.com (W.C.); gbl@cqu.edu.cn (G.L.); 2 Chongqing BOE Optoelectronics Technology Co., Ltd., Chongqing 400714, China; huangzhonghao@boe.com.cn (Z.H.); wuxu@boe.com.cn (X.W.); 3 Chongqing Dongjin Semichem Co., Ltd., Chongqing 400700, China; anjianguo@dongjin.com

**Keywords:** Cu-stacked electrodes, wet etching, profile angle, CD Bias, galvanic effect, thin-film transistors (TFTs)

## Abstract

The electrodes of thin film transistors (TFTs) have evolved from conventional single Cu layers to multi-layered structures formed by Cu and other metals or alloys. Different etching rates of various metals and galvanic corrosion between distinct metals may cause etching defects such as rough or uneven cross-sectional surfaces of stacked electrodes. Therefore, the etching of stacked electrodes faces new challenges. CD Bias and profile angle (PA) are two main performance indicators for the wet etching of TFT electrodes. Adjusting CD Bias and PAs quantitatively and evaluating their stability accurately is crucial to ensure the performance and yield of TFTs. In this work, the bilayer MoNb/Cu-stacked electrodes with different MoNb thicknesses and the MoNb/Cu/MTD triple-layered electrodes were prepared, and the influence of MoNb thickness and stacked structure on the CD Bias and PAs was investigated. It is found that in the H_2_O_2_-based etchant, the order of corrosion potential is E_MTD_ < E_MoNb_ < E_Cu_; both MoNb/Cu and Cu/MTD will form a primary cell with MoNb or MTD as the anodes. The CD Bias and PAs of the MoNb/Cu bilayer structure also increase with MoNb thickness, but those of the MoNb/Cu/MTD triple-layered structure decrease with the introduction of the top MTD film. Finally, regression equations between CD Bias or PA and etching parameters were established based on the results of uniform experiments, and the 95% confidence intervals for CD Bias and PA were proposed after the Monte Carlo simulation. These obtained results provide a basis for quantitative adjustment of CD Bias and PA and precise control of etching stability.

## 1. Introduction

The thin-film transistor (TFT) is the core component of a thin-film transistor liquid crystal display (TFT-LCD) and is generally made up of a gate, a gate insulator (GI), an active layer, and a source-drain (S/D) electrode. Usually, a passivation layer (PVX) covers the TFT device to provide protection, among which 1ITO serves as the pixel electrode connecting with the drain electrode and receives the electrical signal from the TFT device, and 2ITO serves as the common electrode to provide reference potential, and the potential difference between them drives liquid crystal deflection [1,2,3]. With the development of TFT-LCD to larger sizes and higher refresh rates, to avoid signal delay, the electrode materials of TFT gradually adopt Cu or Cu alloys with better conductivity than Al [4,5]. However, due to the poor adhesion between Cu and the glass substrate, the Cu film deposited directly on the glass substrate is prone to peeling off. Therefore, a buffer layer needs to be deposited before Cu film, i.e., the electrode becomes a stacked structure of buffer layer/Cu [6,7,8] (in this paper, the bottom layer is expressed in the front and the top one in the back). In addition, when Cu electrodes are used as SD electrodes, their diffusion into the active layer leads to deterioration of TFT performance [9,10,11,12]. Therefore, the buffer layer below the Cu is also a barrier metal with the ability to block Cu diffusion. Mo, Ti, MoTi, MoAl, Cu alloys, etc., are commonly used as buffer layers [10,13,14,15,16,17]. Cu downward diffusion is blocked by the buffer layer, whereas Cu can still diffuse upward, resulting in deterioration of device performance and stability [18,19,20,21]. Moreover, Cu is easily corroded when it is exposed to the environment or corrosive media [22,23,24]. In order to suppress Cu diffusion along the top and Cu corrosion, the optimal Cu electrode structure is a three-layer stacked structure of buffer layer/Cu/protective layer, with a layer of protective metal on top of the Cu layer [25,26,27,28,29].

**Figure 1:**
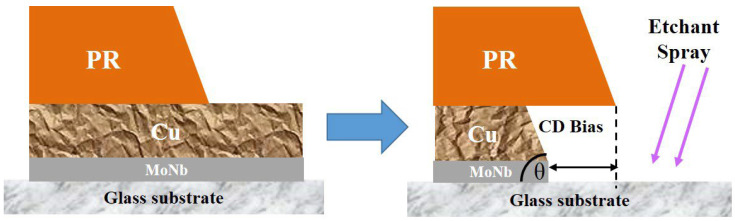
Schematic diagram of CD Bias and profile angle.

The process flow of TFT electrodes mainly includes sputter, photolithography, wet etching, and wet stripping [1,30,31]. The wet etching of an electrode is shown in Figure 1. The metals not protected by photoresist (PR) are etched by a spraying etchant; the isotropic characteristic of wet etching leads to lateral etching. Thus, a trapezoid in the vertical direction of the cross-sectional surface forms, and the acute angle of the trapezoid is called the profile angle (PA) or tape angle (TA) in the TFT industry. Meanwhile, compared with the PR boundary, the etched Cu electrode undergoes a horizontal inward shrinkage, which is known as critical dimension (CD) Bias in the TFT industry. PA and CD Bias are two important indicators to evaluate the performance of wet etching, and their values are mainly determined by process parameters such as etchant composition, etchant concentration, etching time, and temperature. PA and CD Bias are schematically shown in Figure 1.

Different types of products or specifications of TFT products may be manufactured on the same TFT production line, so the corresponding TFT electrode types vary, resulting in various requirements for the etching performance of electrodes. Even the demands for CD Bias and PA size may be completely opposite for different TFTs. For example, in some cases (such as high-resolution TFT products), the electrodes are requested to be finer. Thus, CD bias is required to be as small as possible to facilitate precise control of electrode size. However, in other application scenarios (such as thick Cu etching), the CD bias is expected to be large enough; that is, it is hoped to have a fast-etching rate to shorten the etching time and improve production capacity. As for PA, there may also be conflicting requirements. For instance, a large PA is requisite to ensure low resistance and low parasitic capacitance of the electrodes for certain products, but a low PA is required to improve the coverage of the GI layer on the gate for other products. Therefore, in order to adapt to the etching of different types of electrodes and meet the various requirements of CD Bias and PA, it is urgent to develop wet etching technology on TFT production lines that can quantitatively adjust CD Bias and PA without changing the existing etchant composition.

The etchant for Cu mainly consists of two systems: phosphoric acid and hydrogen peroxide. Among them, the phosphoric acid system is primarily composed of H_3_PO_4_, HNO_3_, and CH_3_COOH, which can damage the IGZO active layer and reduce the stability of TFT devices owing to their strong acidity and corrosion. So, phosphoric acid-based etchant is mainly used for gate etching or SD electrode etching of amorphous silicon TFTs [32,33,34,35]. H_2_O_2_-type etchant has low acidity and a relatively low etching rate and will not damage the active layer. Therefore, H_2_O_2_-type etchant can be applied for electrode etching of amorphous silicon TFT and IGZO TFT, so it is widely employed in mass production [36,37,38].

In our previous work, the CD Bias and PA adjustment techniques of Cu electrode in H_2_O_2_ etchant were investigated, and the regression equations between CD Bias, or PA, and etching parameters (etching time, temperature, Cu ion concentration) were established through which the etching performance can be predicted, and the required etching conditions can be inferred [30,31]. However, these works were limited to MoNb/Cu layered electrodes and did not involve the triple-layered structure of the buffer layer/Cu/protective layer. In addition, although the etching parameters are set to be fixed on the TFT production line, the actual etching time and temperature fluctuate within a small range, and the concentration of Cu ions in the etchant gradually increases, resulting in CD Bias and PA not being constant but oscillating within a range. Therefore, in order to facilitate the judgment of whether CD Bias and PA are qualified, it is necessary to provide a confidence interval. Exceeding the upper and lower limits of this interval indicates that the sample quality is unqualified.

Therefore, in the present work, the MoNb/Cu double-layered electrodes with different MoNb thicknesses and the MoNb/Cu/MTD triple-layered electrodes were prepared on the TFT 8.5-generation (G) production line, and the influence of MoNb thickness and layered structure on the forming process of profile and etching performance (CD Bias and PA) was discussed. The contact angle (CA) and the corrosion potential (E_corr_) of Cu, MoNb, and MTD were measured. The influence of etching conditions on the etching performance of the three stacked electrodes was investigated through a uniform experiment, and regression equations of CD Bias or PA with etching parameters were established. Subsequently, Monte Carlo simulation was used to generate random 100,000 etching parameters to obtain 100,000 sets of CD Bias and PA data. Then, the obtained CD Bias and PA data were statistically analyzed to determine their statistical distributions and identify their 95% confidence intervals. At last, the upper and lower control limits of CD Bias and PA for different kinds of electrodes are determined. The establishment of a confidence interval will provide a judgment basis for sampling tests for the etching production line, which is crucial for etching quality control. This study can provide guidance for ensuring TFT yield and improving product performance under mass production conditions.

## 2. Materials and Methods

### 2.1. Preparation of Electrode Samples with Different Stacked Structures

**Figure 2:**
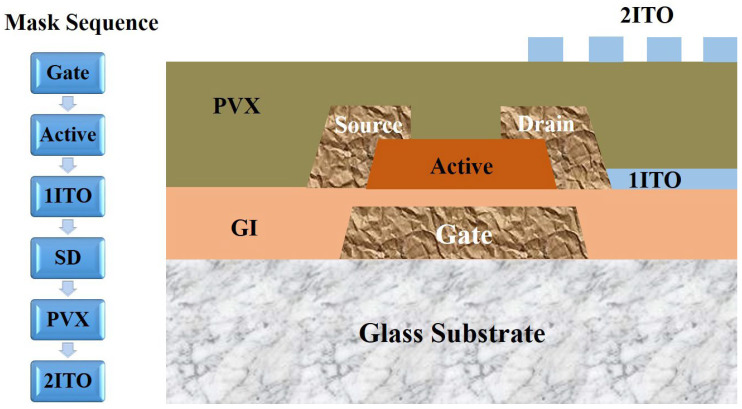
Schematic diagram of TFT device structure.

**Figure 3:**
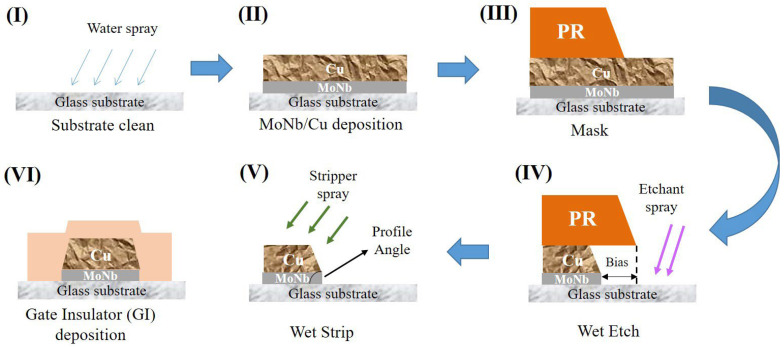
Schematic diagram of the fabrication process of the gate electrode.

Electrode samples with different stacked layer structures for TFT devices were prepared on the 8.5-G TFT production line of Chongqing BOE Optoelectronics Technology Co. Ltd. The TFT structure is shown in Figure 2, where a gate, a GI, an a-Si active layer, 1ITO, an S/D electrode, a PVX layer, and 2ITO were sequentially deposited on a glass substrate to form a device. The fabrication process of the gate electrode is displayed in Figure 3, where the glass substrate was successively subjected to cleaning (Initial Clean, DMS, Yongin, Republic of Korea), deposition (2400D, ULVAC, Chigasaki, Japan), photolithography (H763, Canon, Tokyo, Japan), etching (Wet Etch, DMS, Yongin, Republic of Korea), and wet stripping (Wet Strip, DMS, Yongin, Republic of Korea) to fabricate the patterned electrodes. After that, a layer of GI was deposited.

In this paper, three single-layer films of MoNb (30 nm), Cu (300 nm), and MTD (MoNiTi alloy, 30 nm) layers; two double-stacked layers of MoNb/Cu (15/300 nm) and MoNb/Cu (30/300 nm); and one triple-stacked layer structure electrode of MoNb/Cu/MTD (15/300/20 nm) were deposited on glass substrate using a sputtering method, and these stacked layer structure electrodes were sequentially subjected to photolithography and wet-etching (H_2_O_2_ etchant) to form electrode patterns.

Wet etching was to be worked as two sets of experiments. In the first set, the etching parameters were fixed to examine the effect of electrode structure on CD Bias and PA. The etching parameters (temperature, Cu Cu ion concentration, etch time) were 30 °C, 2000 ppm, and 50 to 100 s, respectively. In the second group, a uniform experiment was designed to investigate the effects of etching parameters on CD Bias and PA of three stacked electrodes. The etching time, temperature, and Cu ion concentration were treated as independent variables, and CD Bias and PA were considered as dependent variables, and the combinations of etching parameters are shown in Table 1.

### 2.2. Measurement of Contact Angle and Electrochemical Performance

The contact angle (CA) of three single-layer films, MoNb, Cu, and MTD, deposited on glass substrates, was measured with a high-speed camera (Phantom v7.3 Vision Research, Wayne, NJ, USA). Based on the contact angle measurements, the adhesion of Cu or MTD to the PR was indirectly characterized.

After completing the above tests, the three monolayer samples of MoNb, Cu, and MTD were cut into 2 cm × 2 cm small pieces, which were used as the working electrodes of the electrochemical workstation (CHI660E, Shanghai Chenhua Instrument Co., Shanghai, China), and the saturated calomel electrode and platinum sheet (2 cm × 2 cm) were used as the reference and auxiliary electrodes, respectively. The polarization curves of MoNb, Cu, and MTD in the H_2_O_2_ etchant were tested at a scanning rate of 1 mV/s to clarify the respective corrosion potentials and then to identify the galvanic effects between MoNb/Cu, Cu/MTD, and MoNb/MTD.

### 2.3. Characterization of Films and Electrode Samples

SEM microscope (QQSEM-01, Hitach, Tokyo, Japan) was used to observe the cross-sectional morphology of the samples of the above two parts of the etching experiments, measuring the CD Bias and PA. In the first part of the etching experiments, the effect of etching time on the CD Bias and PA was investigated, and the differences in CD Bias and PA of the three kinds of stacked electrodes were compared to clarify the regulation effect of the thickness of the bottom MoNb and the MTD on the etching performance.

In the second part of the etching experiments, regression analysis was used to establish the regression equations of the etching parameters (etching time, temperature, and Cu ion concentration) with respect to CD Bias and PA. In addition, the model error (S) of each regression equation was also confirmed through regression analysis. From the statistical characterization, the regression equation is satisfied with a mean of 0 and a standard deviation of S data fluctuations. Therefore, a normal distribution with mean 0 and standard deviation S is used as the benchmark, and 100,000 sets of random numbers under this distribution are generated as the model error of the regression equation.

Based on the etching parameters of a specific product in the TFT production line (30 °C, 85 s, and Cu ion concentration in the range of 1000~3000 ppm), the actual etching parameters of the product over a long period of time were collected, and its statistical distribution was identified by the analysis of Minitab software (Minitab 18). In accordance with the statistical distribution of the etching parameters, 100,000 sets of etching parameters were randomly generated by Minitab software, and then corresponding 100,000 sets of CD Bias and PA data related to different stacked electrodes will be calculated by the regression equation, respectively. Then, the PA and CD Bias values obtained from calculations were summed with the respective regression equation errors, and the summed values were used as the final data. After that, the statistical distributions of the obtained CD Bias and PA data can be determined, and the 95% confidence intervals can be specified. Its upper and lower limits were regarded as the upper and lower control limits for CD Bias and PA.

## 3. Results and Discussion

### 3.1. Contact Angles of Three Metal Film Layers

The CAs of Cu, MoNb, and MTD with three liquids with different polarities, water (H_2_O), ethylene glycol ((CH_2_OH)_2_), and diiodomethane (CH_2_I_2_), were measured, among which the first two are polar, but the last one is non-polar. The measurement data are shown in Table 2; the CAs of Cu, MoNb, and MTD were tested in water at 78°, 35°, and 50°, respectively. The CAs corresponding to ethylene glycol for Cu, MoNb, and MTD are 54°, 21°, and 36°, respectively, which are in the same trend of ranking relationship of CAs as those corresponding to H_2_O. The CAs of Cu, MoNb, and MTD corresponding to diiodomethane were 37°, 33°, and 35°, respectively, with small variations in the values, but the trends of the CAs ranking relationship are still consistent with those corresponding to water.

It indicates that Cu is relatively hydrophobic, but MoNb and MTD are relatively hydrophilic. In the stacked electrodes, MoNb acts as a buffer layer and is always at the bottom, while Cu and MTD are at the top of the stack and will directly contact PR. Since the PR is oily and hydrophobic, the CA difference in Cu, MoNb, and MTD between PR is MoNb-PR > MTD-PR > Cu-PR. M. L. Tan et al. reported that the greater the CA difference between the two materials, the lower the interfacial bonding strength [39]. So it can be inferred that the interfacial adhesion strength between Cu, MoNb, and MTD between PR is MoNb-PR < MTD-PR < Cu-PR, which means the adhesion between Cu and the PR is larger than that of the MTD/PR and MoNb/PR, which will cause different PA or CD bias for stacked electrode structures with various top layers contacting PR.

### 3.2. Electrochemical Properties of Three Metal Layers

**Figure 4:**
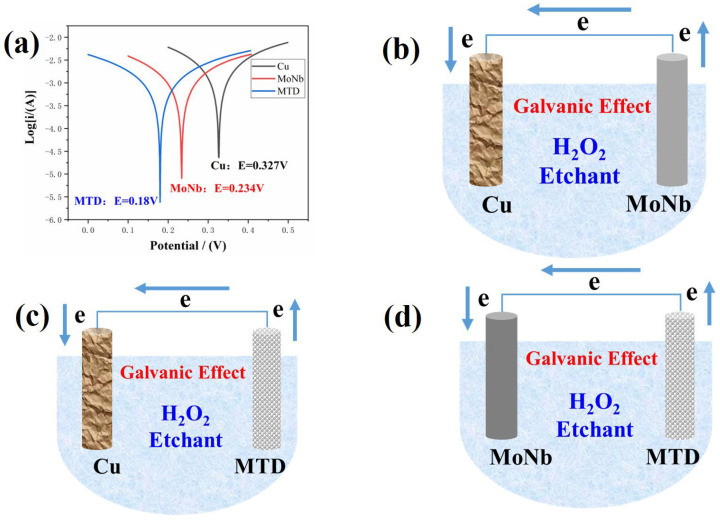
(**a**) Polarization curves of Cu, MoNb, and MTD; Schematic primary cells of (**b**) MoNb/Cu, (**c**) Cu/MTD, and (**d**) MoNb/MTD.

The polarization curves of Cu, MoNb, and MTD, three metals examined in the H_2_O_2_-based etchant, are displayed in Figure 4a, and their corrosion potentials (E_corr_) are 0.327 V, 0.234 V, and 0.18 V, respectively, meaning sorting by easiest to corrode: MTD > MoNb > Cu. So, a galvanic effect will occur in the MoNb/Cu, Cu/MTD, and MoNb/MTD stacked electrodes, and the corresponding primary cells are exhibited in Figure 4b,c,d, respectively. As shown in Figure 4b, in the MoNb/Cu stacked electrode, due to the lower E_corr_ of MoNb, MoNb acts as the anode to lose electrons and accelerates corrosion, and Cu acts as the cathode and receives electrons, so the Cu corrosion is suppressed. Similarly, a primary cell will form in the Cu/MTD stack structure, as shown in Figure 4c, in which MTD acts as an anode, and Cu acts as a cathode to receive electrons. In the MoNb/MTD stack, as shown in Figure 4d, MTD acts as the anode while MoNb acts as the cathode.

### 3.3. Influence of Etching Time on Etching Performance of Stacked Electrodes

**Figure 5:**
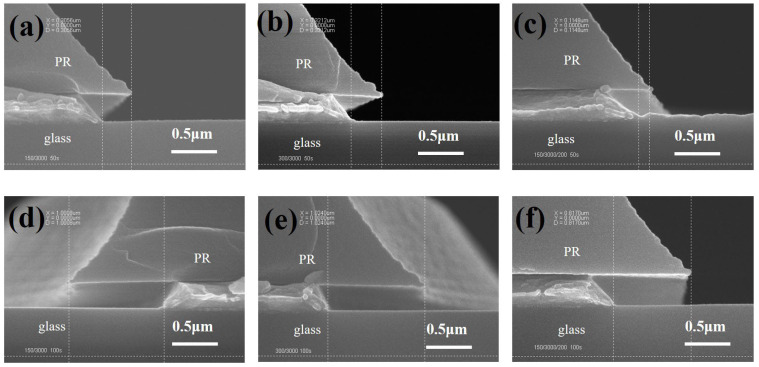
Cross-sectional morphology of the three stacked electrodes after etching for different times: (**a**–**c**) 50 s; (**d**–**f**) 100 s; (**a**,**d**) MoNb/Cu (15/300 nm); (**b**,**e**) MoNb/Cu (30/300 nm); (**c**,**f**) MoNb/Cu/MTD (15/300/20 nm). The dotted lines are generated by the measurement software affiliated with the SEM equipment and are used to measure the CD Bias. One vertical dotted line is based on the profile angle of the PR, the other vertical dotted line is based on the profile angle of the electrode, and the horizontal distance between the two vertical dotted lines is the CD Bias.

In the mass production etching conditions, the concentration of Cu ions in the etchant gradually increases with an increase in flow sheets. Cu ions promote the decomposition of H_2_O_2_, and the etching will be accelerated when the concentration of Cu ions increases. The etching temperature was kept at 30 °C, and the Cu ion concentration in the etchant was kept at 2000 ppm, and the three kinds of stacked-structure electrodes, MoNb/Cu (15/300 nm), MoNb/Cu (30/300 nm), and MoNb/Cu/MTD (15/300/20 nm), were etched for 50–100 s, respectively. The cross-sectional morphology of the three kinds of stacked electrodes etched for 50 s and 100 s are shown in Figure 5a–c and d–f, respectively. It can be seen that when etching for 50 s, MoNb/Cu (15/300 nm) and MoNb/Cu (30/300 nm) have smooth etching cross sections and no etching residue, while MoNb/Cu/MTD (15/300/20 nm) has smooth etching cross sections but has etching residue. When the etching time is increased to 100 s, the cross sections of all three stacked layers are smooth and without etching residue.

**Figure 6:**
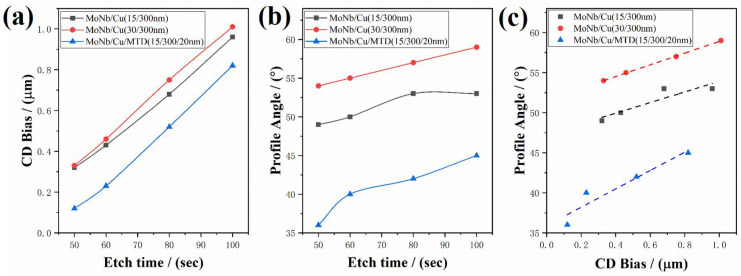
Effect of etching time on etching performance of the three stacked electrodes. (**a**) The dependence of CD Bias on etching time, (**b**) the dependence of PA on etching time, and (**c**) the relationship between CD Bias and PA.

The relationships of CD Bias and PA with different etching times are shown in Figure 6a,b, respectively. It can be seen that both CD Bias and PA increase with increasing etching time. In the same etching conditions, the order of CD Bias sorted by length is MoNb/Cu (30/300 nm) > MoNb/Cu (15/300 nm) > MoNb/Cu/MTD (15/300/20 nm). Similarly, the order of PA according to angle magnitude is MoNb/Cu (30/300 nm) > MoNb/Cu (15/300 nm) > MoNb/Cu/MTD (15/300/20 nm). It can be found that both CD Bias and PA increase with an increase in bottom MoNb thickness but decrease after the introduction of MTD on the top. As shown in Figure 6c, there is a positive correlation between CD Bias and PA.

### 3.4. Effect of Stacking Structure on Formation Process of Profile and Etching Performance

The effect of the bottom MoNb thickness and the introduction of MTD on the top on the etching performance of the electrodes of the stacked structure is related to the formation of the profile angle during the etching process.

**Figure 7:**
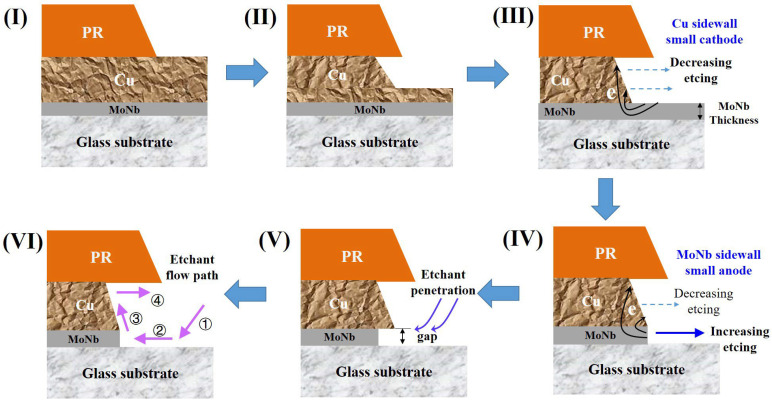
The formation of the profile angle of MoNb/Cu bilayer-stacked electrodes in the etching process.

The etching process of the double-stacked MoNb/Cu electrode is shown in Figure 7. When the etching proceeds to stage III, the Cu film not covered by PR is completely etched in the vertical thickness direction; thus, the bottom MoNb is exposed. The MoNb on the bottom and the Cu on the sidewall form a primary cell, in which MoNb acts as the anode to lose electrons, and the Cu sidewall acts as the cathode to receive electrons, so the etching of the Cu sidewall is suppressed. Owing to the large area of the MoNb and a small area of Cu on the sidewall, the MoNb/Cu primary cell is one with a large anode|small cathode. Thus, the electron concentration received at the small-area Cu cathode position is high, causing an obvious inhibition of Cu lateral etching. As the bottom MoNb layer becomes thicker, its required etching time gets longer, which means that the duration of the large anode|small cathode primary cell increases, the inhibition of lateral etching of Cu becomes stronger, and finally, the PA increases.

When the etching proceeds to stage IV, the bottom MoNb not covered by Cu is completely etched in the thickness direction; the MoNb sidewall and Cu sidewall are a small anode | large cathode primary cell in which the bottom MoNb acts as an anode and loses electrons to accelerate the etching. As the etching proceeds to the stage, the bottom MoNb shrinks inward due to the accelerated etching and forms a gap between the Cu and the substrate. As the bottom MoNb thickness increases, the gap widens, and the etchant penetrates more easily from the bottom, so the degree of etching is enhanced, which ultimately leads to an increase in CD Bias.

When the etching continues to stage VI, the etchant may form a reflux, as displayed in Figure 7. The fresh etchant reaches the substrate first, then contacts the bottom of the electrode, and subsequently arrives at the top of the electrode along the slope of the electrode. The entire etchant reflux process is accompanied by the cooling of the etching solution and the decomposition of H_2_O_2_, so the etching ability gradually decreases, i.e., the etching rate at the bottom of the electrode is greater than that at the top of the electrode. Secondly, the PR on the top of the electrode will hinder the flow of the etchant, and the replacement rate of the etchant on the top is lower than that on the bottom, which further causes a decrease in the etching rate at the top of the electrode. When the etching time is prolonged, the electrode etching increases, so the CD Bias becomes larger. Meanwhile, the difference in the etching degree between the top and bottom of the electrode caused by the prolonged time is amplified, so the bottom etching rate gets much faster, resulting in a gradual increase in PAs.

**Figure 8:**
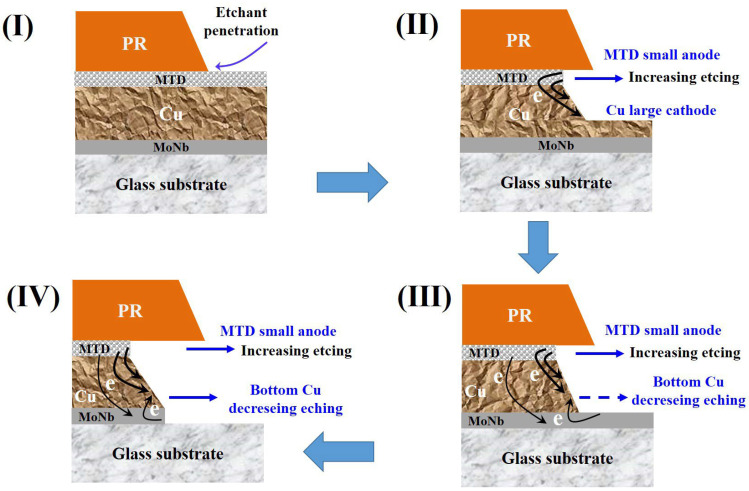
The formation of the profile angle of MoNb/Cu/MTD triple-stacked electrodes in the etching process.

The etching process of the triple-stacked MoNb/Cu/MTD electrode is shown in Figure 8. In this triple-stacked layer, the introduction of MTD at the top increases the amount of metal required for etching. Thus, the H_2_O_2_ consumption increases, resulting in a decrease in the etching rate under the same etching conditions. Correspondingly, the CD Bias in the etched MoNb/Cu/MTD is smaller than that of MoNb/Cu. During the etching process of MoNb/Cu/MTD, the etchant reflux and the PR may hinder the etchant update at the top of the electrode. Ultimately, the PA of MoNb/Cu/MTD tends to increase as the etching time increases.

As the adhesion between MTD and PR is lower than that of Cu, the etchant is more likely to intrude along the MTD/PR interface during the etching process in stage I, as shown in Figure 8, causing an increased lateral etching at the top of the electrode. As a result, the PA of MoNb/Cu/MTD is lower than that of MoNb/Cu under the same etching conditions.

In addition, the MTD can form a primary cell with Cu and MoNb to reduce the PA by galvanic effect. In stage II, the MTD not covered by PR is completely etched, and the exposed Cu and MTD sidewalls will form a small anode|large cathode-type primary cell, in which the MTD as an anode accelerates the corrosion and the lateral etching of the electrode increases.

When the Cu not covered by PR is all etched away in the vertical direction, the etching process reaches stage III in Figure 8, where a large area of bottom MoNb is exposed. MoNb sidewalls are all exposed; the etching arrives in Stage IV. During stages III and IV, three primary cells may occur: MoNb/Cu, Cu/MTD, and MoNb/MTD. In the MoNb/MTD primary cell, Cu acts as a wire connecting the MTD sidewall and bottom MoNb.

Suppose we define the electric potential of a primary cell (E_cell_) as the E_corr_ difference in the cathode and anode, i.e., E_cell_ = E_corr-cathode_ − E_corr-anode_. According to the E_corr_ results obtained from the polarization curves of Cu, MoNb, and MTD in Figure 4: E_corr-Cu_ = 0.327 V, E_corr-MoNb_ = 0.234 V, and E_corr-MTD_ = 0.18 V; then, E_cell-MTD|Cu_ = 0.147 V > E_cell-MoNb|Cu_ = 0.093 V > E_cell-MTD/MoNb_ = 0.054 V, indicating that the electrons transmitted between the anode and cathode sorted by the number in descending order are Cu/MTD > MoNb/Cu > MoNb/MTD, meaning the transmitted electrons among the three primary cells are mainly from the Cu. The number of electrons transmitted is indicated by the boldness of the arrows in Figure 8.

During stages III and IV in Figure 8, both in the Cu/MTD primary cell and the MoNb/MTD primary cell, the top MTD always acts as an anode to accelerate the lateral etching. Also, in the same stage, with the MoNb/Cu primary cell, the Cu near the bottom MoNb acts as a cathode, and the etching is suppressed, i.e., the lateral etching of the bottom Cu is decelerated. The lateral etching at the top of the electrode is higher than that at the bottom, and eventually, PA is lower.

To sum up, due to the relatively low PR adhesion and galvanic effects of MTD, the top MTD in the MoNb/Cu/MTD stacked structure plays the role of sacrificial anode, causing the lateral etching in the MoNb/Cu/MTD stacked electrode to get greater, resulting in the PA of the MoNb/Cu/MTD stacked electrode being smaller than that of the MoNb/Cu.

### 3.5. Etching Performance of Three-Stacked Electrodes Under Different Etching Conditions

The influence of etching conditions on the etching performance of the three stacked electrodes was investigated by carrying out a uniform experimental method, in which the etching temperature, etching time, and Cu ion concentration were regarded as independent variables, CD Bias and PA as dependent variables, and the U_12_ (6×4×3) table was chosen and designed. According to the designed experimental scheme, three kinds of stacked structures, MoNb/Cu (15/300 nm), MoNb/Cu (30/300 nm), and MoNb/Cu/MTD (15/300/20 nm), were etched, and their CD Bias and PAs data are listed in Table 1. It can be seen that under the same etching conditions, the CD Bias and PA tend to increase with the increase in MoNb thickness but decrease with the introduction of top MTD, which is consistent with the experimental results in Figure 6.

**Figure 9:**
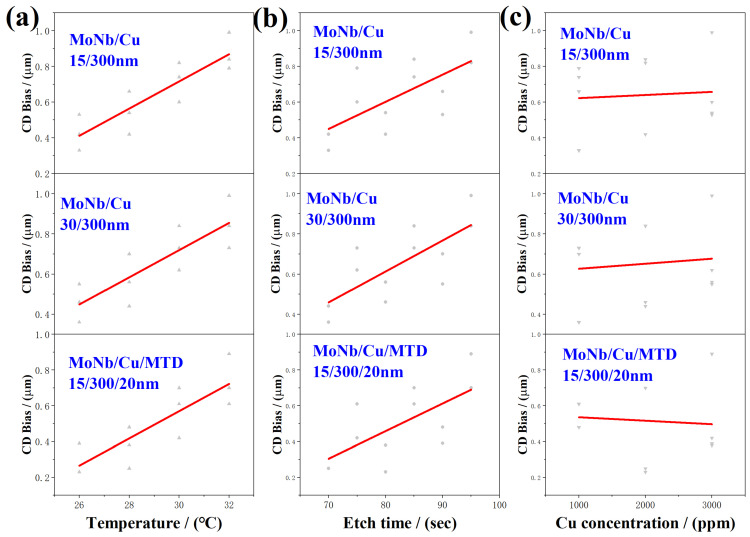
The relationship between CD Bias and etching parameters obtained from uniform experiments: (**a**) etching temperature (Regular triangles); (**b**) etching time (Black circles); (**c**) Cu ion concentration (Inverted Triangle).

**Figure 10:**
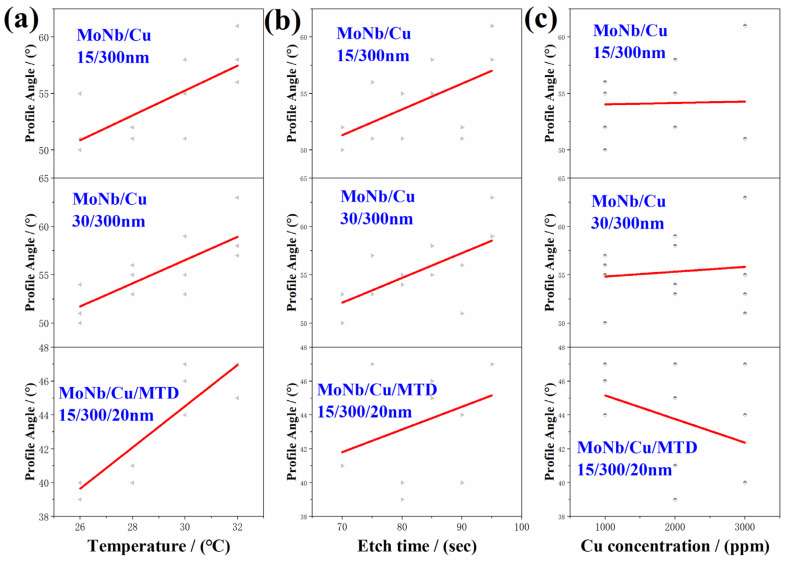
The relationship between PA and etching parameters obtained from uniform experiments: (**a**) etching temperature (right-deviation triangles), (**b**) etching time (left-inclined triangles), and (**c**) Cu ion concentration (white circles).

According to the experimental results in Table 1, the obtained relationships of CD Bias and PA with etching conditions were displayed in Figure 9 and Figure 10, respectively. It is evident from Figure 9a,b that CD Bias increases with either an increase in etching temperature or etching time. From Figure 9c, it can be seen that with the increase in Cu ion concentration, the CD Bias of each stacked electrode did not show obvious changes, indicating that the effect of Cu ions on the CD Bias is significantly smaller than the etching time and temperature, so its effect can be ignored.

The influence of etching parameters on the PA of three stacked electrodes is shown in Figure 10. Owing to the combined effect of the etchant reflux and the PR hindering the etchant update at the top of the electrode, the difference in etching rate between the bottom and the top of the electrode will become greater with stronger etching. As the etching temperature and etching time increase, the etching of the electrode gets more intense. Thus, PAs enhance with the etching temperature or etching time, as shown in Figure 10a,b, respectively.

As illustrated in Figure 10c, the PAs of the MoNb/Cu electrode remained stable with increasing Cu ion concentration, but those of the MoNb/Cu/MTD electrode showed a decreasing trend. As discussed in Section 3.4 and shown in Figure 8, during the etching process of MoNb/Cu/MTD electrodes, except for stage I (the etching of a single layer top MTD), the remaining etching stages are accompanied by galvanic effects of MTD, and the sidewall of the MTD as a small anode will accelerate the lateral etching. When the concentration of Cu ions in the etchant increases, the resistance of the etchant decreases, the galvanic current in the electrochemical effect is therefore increased, and the galvanic effect is further enhanced; the etching rate of the MTD is further aggravated, i.e., the electrode’s lateral etching degree on the top is further increased, which is manifested as a decrease in the PA of the electrode.

Based on the experimental data listed in Table 2, the regression equations of CD Bias and PA changing with the etching parameters can be obtained by eliminating the non-significant influencing factors. The regression equation is shown as follows.

CD Bias (µm) = A × Temp + B × Time + C(1)

PA (°) = D × Temp + E × Time + F × C_Cu_ + G (2)

Temp, Time, and C_Cu_ denote etching temperature (°C), etching time (s), and Cu ion concentration (ppm), respectively. And A, B, D, E, and F are coefficients, and C and G are two constants. The four coefficients and two constants for CD Bias and PA regression equations are summarized in Table 3. In addition, the adjusted R^2^ corresponding to each regression equation set is given in Table 3. For the regression equation, the closer R^2^ is to 100%, the more accurate the model is. The difference in the regression equation R^2^ is characterized by the model error S. From the statistical characterization, the regression equation is satisfied with a mean of 0 and a standard deviation of S data fluctuations.

### 3.6. Evaluation of Etching Performance Stability

**Figure 11:**
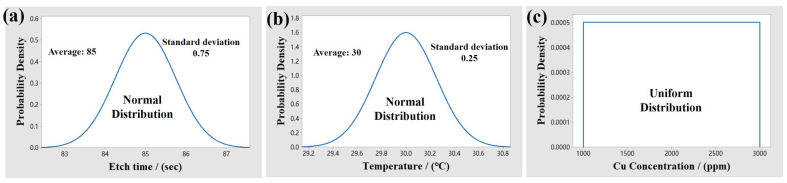
Statistical distribution model of actual etching parameters: (**a**) etching time; (**b**) etching temperature; (**c**) Cu ion concentration.

The etching performance stability was evaluated by Monte Carlo simulation. The etching time and temperature of the Cu electrode of a specific product are, respectively, set to be 85 s and 30 °C, and the concentration of Cu ions in the etchant gradually increases with the number of etched products in the production line. The actual etching parameters of this product for a long period were collected and statistically analyzed. As displayed in Figure 11a,b, it is found that both the actual etching time and temperature as a whole follow a normal distribution, which has a mean value of 85 s, a standard deviation of 0.75 s, a mean value of 30 °C, and a standard deviation of 0.25 °C, respectively. However, as shown in Figure 11c, the Cu ion concentration obeys a uniform distribution as a whole.

**Figure 12:**
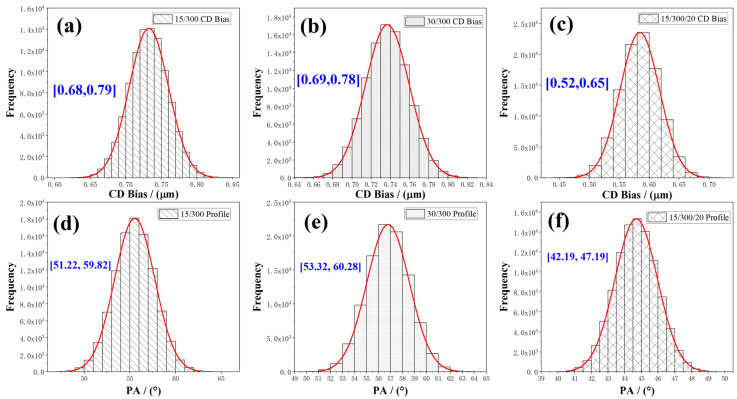
The normal distribution model of CD Bias and PA was obtained by Monte Carlo simulation. (**a**–**c**) CD Bias; (**d**–**f**) PA; (**a**,**d**) MoNb/Cu (15/300 nm); (**b**,**e**) MoNb/Cu (30/300 nm); (**c**,**f**) MoNb/Cu/MTD (15/300/20 nm).

According to the evaluation method described in Section 2.3, the CD Bias and PA data of the three stacked electrodes obtained for 100,000 groups of etching parameters randomly generated through Monte Carlo simulation were statistically analyzed, and the statistical model of CD Bias and PA was given in Figure 12. It shows that the CD Bias and PAs of each electrode conformed to normal distribution.

From the normal distribution model of CD Bias and PA, the mean, standard deviation, and 95% confidence interval information can be obtained through statistical analysis. The 95% confidence interval of CD Bias and PA is manifested in Figure 12.

For CD Bias and PA, rounding and retaining valid data are treated. From Figure 12a, the 95% confidence interval of CD Bias for the MoNb/Cu (15/300 nm) electrode is [0.68, 0.79], implying that the probability that the CD Bias distributes in the range of 0.68–0.79 µm is 95%. If 0.68 µm and 0.79 µm are, respectively, set as the lower and upper limits to control the etching quality for 15/300 MoNb/Cu electrodes, the samples with CD Bias data falling within the confidence interval are qualified products, and the etching process is stable and reliable. If the sampled CD Bias data fall outside the confidence interval, it can be considered that the etching process is abnormal, and the technological process or equipment troubleshooting is required. The 95% confidence intervals for PA distributions can be determined similarly.

The 95% confidence intervals for CD Bias are [0.68 µm, 0.79 µm], [0.69 µm, 0.78 µm], and [0.52 µm, 0.65 µm]; for PA, they are [51.22°, 59.82°], [53.32°, 60.28°], and [42.19°, 47.19°] for MoNb/Cu (15/300 nm), MoNb/Cu (30/300 nm), and MoNb/Cu/MTD (15/300/20 nm), respectively.

This method can be used to evaluate etch stability for the remaining mass production conditions.

## 4. Conclusions

In this paper, the wet etching process of MoNb/Cu electrodes with different MoNb thicknesses and MoNb/Cu/MTD stacked electrodes was investigated, the effect of MoNb thicknesses and stacked structures on the CD bias and PA was identified, and the techniques to quantitatively adjust the CD Bias and PA without changing the components of the etchant were proposed. The main conclusions are as follows:

1. The contact angles of Cu and MTD with H_2_O are 78° and 50°, respectively. The interfacial adhesion between MTD and PR is weaker than that of Cu and PR, indicating that the etchant is more likely to penetrate along the MTD/PR interface to enhance the lateral etching.

2. In the H_2_O_2_-based etchant, the corrosion potential of Cu, MoNb, and MTD is 0.327 V, 0.234 V, and 0.18 V, respectively. MoNb/Cu, Cu/MTD, and MoNb/MTD can form a primary cell. In the Cu/MTD primary cells, MTD acts as an anode and accelerates etching, and Cu as a cathode, its etching is inhibited. In the MoNb/Cu primary cell, Cu acts as a cathode due to the high corrosion potential; Cu etching is inhibited, and bottom MoNb etching is accelerated.

3. As the bottom MoNb thickness in the MoNb/Cu electrode increases, both CD Bias and PA increase. In the etching process of the MoNb/Cu dual-layer electrode, a large-area bottom MoNb anode/small-area Cu sidewall cathode primary cell will occur. The thicker MoNb needs longer etching time, causing the lateral etching of MoNb to be stronger and PA larger, and the gap between MoNb and Cu to be bigger, and more etchant infiltrating along the bottom leads to an enhanced bottom etching, ultimately resulting in a larger CD Bias.

4. Both CD Bias and PA decrease after the introduction of MTD on the top of the MoNb/Cu structure. The addition of MTD leads to an increase in the consumption of H_2_O_2_ in the etchant and a decrease in the etching rate, so CD Bias decreases. Owing to the relatively poor PR adhesion and galvanic effects of MTD, the top MTD in the MoNb/Cu/MTD stacked structure plays the role of sacrificial anode, causing its lateral etching to get greater, resulting in the PA of the MoNb/Cu/MTD stacked electrode being smaller than that of the MoNb/Cu.

5. The regression equations of CD Bias and PA changing with the etching parameters were obtained. The 95% confidence intervals for CD Bias and PA of MoNb/Cu (15/300 nm), MoNb/Cu (30/300 nm), and MoNb/Cu/MTD (15/300/20 nm) are provided. Though comparing the value of CD Bias and PA falling within the confidence interval or not, whether an etched product is qualified or not and whether the etching process is stable and reliable or not can be judged accurately. This method provides guidance for quality control of wet etching of Cu electrodes, which is very helpful in improving TFT yield and device performance.

## Figures and Tables

**Table 1:** Uniform experimental schemes and results for the etching of three stacked electrodes.

Num	Tem (°C)	Time (S)	C_Cu_ (ppm)	15/300		30/300		15/300/20	
				Bias/μm	PA/°	Bias/μm	PA/°	Bias/μm	PA/°
U1	26	70	1000	0.33	50	0.36	50	Reamin	Reamin
U2	28	70	2000	0.42	52	0.44	53	0.25	41
U3	30	75	3000	0.60	51	0.62	53	0.42	44
U4	32	75	1000	0.79	56	0.73	57	0.61	47
U5	26	80	2000	0.42	55	0.46	54	0.23	39
U6	28	80	3000	0.54	51	0.56	55	0.38	40
U7	30	85	1000	0.74	55	0.73	55	0.61	46
U8	32	85	2000	0.84	58	0.84	58	0.70	45
U9	26	90	3000	0.53	51	0.55	51	0.39	40
U10	28	90	1000	0.66	52	0.70	56	0.48	44
U11	30	95	2000	0.82	58	0.84	59	0.70	47
U12	32	95	3000	0.99	61	0.99	63	0.89	47

**Table 2:** Contact angles of Cu, MoNb, and MTD with water, ethylene glycol, and diiodomethane [1].

	H_2_O	(CH_2_OH)_2_	CH_2_I_2_
Cu	78°	54°	37°
MoNb	35°	21°	33°
MTD	50°	36°	35°

**Table 3:** Coefficients and constants for CD Bias and PA regression equations.

Categories	Coefficients	MoNb/Cu 15/300			MoNb/Cu 30/300			MoNb/Cu/MTD 15/300/20		
		Parameters	R^2^	S	Coefficients	R^2^	S	Coefficients	R^2^	S
CD Bias (μm)	A (μm/°C)	0.06528	98.87%	0.021	0.05608	99.27%	0.016	0.07091	98.44%	0.026
	B (μm/s)	0.010724			0.011583			0.013504		
	C	−2.1377			−1.9303			−2.691		
PA (°)	D (°/°C)	0.936	61.79%	2.187	1.012	76.40%	1.749	1.111	87.23%	1.110
	E (°/s)	0.1644			0.1877			0.1129		
	F (°/ppm)	0			0			−0.000972		
	G	13.47			10.49			3.71		

## Data Availability

The data presented in this study are available on request from the corresponding author.
